# Nicotine Acts on Growth Plate Chondrocytes to Delay Skeletal Growth through the α7 Neuronal Nicotinic Acetylcholine Receptor

**DOI:** 10.1371/journal.pone.0003945

**Published:** 2008-12-16

**Authors:** Atsuo Kawakita, Kazuki Sato, Hatsune Makino, Hiroyasu Ikegami, Shinichiro Takayama, Yoshiaki Toyama, Akihiro Umezawa

**Affiliations:** 1 Department of Reproductive Biology, National Institute for Child Health and Development, Tokyo, Japan; 2 Department of Orthopaedic Surgery, Keio University School of Medicine, Tokyo, Japan; 3 Department of Orthopaedic Surgery, National Center for Child Health and Development, Tokyo, Japan; University of Giessen Lung Center, Germany

## Abstract

**Background:**

Cigarette smoking adversely affects endochondral ossification during the course of skeletal growth. Among a plethora of cigarette chemicals, nicotine is one of the primary candidate compounds responsible for the cause of smoking-induced delayed skeletal growth. However, the possible mechanism of delayed skeletal growth caused by nicotine remains unclarified. In the last decade, localization of neuronal nicotinic acetylcholine receptor (nAChR), a specific receptor of nicotine, has been widely detected in non-excitable cells. Therefore, we hypothesized that nicotine affect growth plate chondrocytes directly and specifically through nAChR to delay skeletal growth.

**Methodology/Principal Findings:**

We investigated the effect of nicotine on human growth plate chondrocytes, a major component of endochondral ossification. The chondrocytes were derived from extra human fingers. Nicotine inhibited matrix synthesis and hypertrophic differentiation in human growth plate chondrocytes in suspension culture in a concentration-dependent manner. Both human and murine growth plate chondrocytes expressed alpha7 nAChR, which constitutes functional homopentameric receptors. Methyllycaconitine (MLA), a specific antagonist of alpha7 nAChR, reversed the inhibition of matrix synthesis and functional calcium signal by nicotine in human growth plate chondrocytes in vitro. To study the effect of nicotine on growth plate in vivo, ovulation-controlled pregnant alpha7 nAChR +/− mice were given drinking water with or without nicotine during pregnancy, and skeletal growth of their fetuses was observed. Maternal nicotine exposure resulted in delayed skeletal growth of alpha7 nAChR +/+ fetuses but not in alpha7 nAChR −/− fetuses, implying that skeletal growth retardation by nicotine is specifically mediated via fetal alpha7 nAChR.

**Conclusions/Significance:**

These results suggest that nicotine, from cigarette smoking, acts directly on growth plate chondrocytes to decrease matrix synthesis, suppress hypertrophic differentiation via alpha7 nAChR, leading to delayed skeletal growth.

## Introduction

Though detrimental effects of cigarette smoking to the human body have been widely demonstrated, the effects on endochondral ossification are not well understood. Epidemiologically, maternal smoking reduces the height of newborns [1]–[5]. However, there are controversial views regarding the mechanisms behind delayed skeletal growth caused by cigarette smoking. The socioeconomic status of smoking mothers [6], [7], deficient maternal diet [8], chronic hypoxia caused by carbon monoxide [9], impaired placental size and function, and decreased blood flow of placenta caused by nicotine [10] have all been reported as a possible causal factors responsible for reduction in height of newborns. Conversely, it has also been reported that socioeconomic status [11], maternal diet [12], and hypoxia are not responsible for the cause of delayed skeletal growth. Research suggests that smoking not only reduces body length but also brings ossification retardation in the rat smoking model [13]. Moreover, smoking delays chondrogenesis in a mouse model of fracture healing [14]. Cigarette smoking, thus, adversely affects endochondral ossification somehow during the course of skeletal growth and repair in animal models.

Among a multitude of chemicals and physiological functions arising from cigarette smoking, nicotine is one of the leading candidates for causing small newborns. Epidemiologically, nicotine content in cigarette is related to reduced birth length in humans [15]. However, the possible mechanism of delayed skeletal growth caused by nicotine remains unclarified. In this study, we investigated the effect of nicotine on growth plate chondrocytes, the principle component of endochondral ossification. In the last decade, localization of neuronal nicotinic acetylcholine receptor (nAChR), a specific receptor of nicotine, has been widely detected in non-excitable cells [16]. Therefore, we hypothesized that nicotine affect growth plate chondrocytes directly and specifically through nAChR to delay skeletal growth. We here demonstrate that nicotine affected growth plate chondrocytes through alpha7 nAChR to decrease matrix synthesis and to suppress hypertrophic differentiation, thereby delaying skeletal growth.

## Results

### Detection and localization of nAChR in growth plate chondrocytes

**Figure 1:**
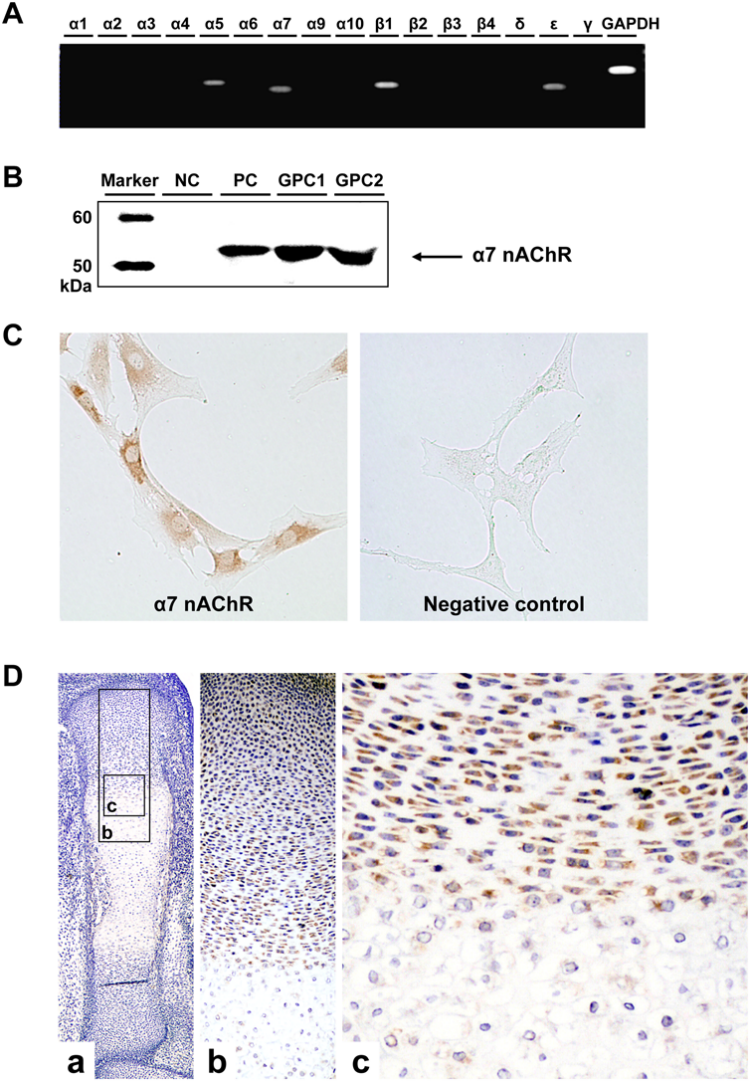
Detection and localization of nAChR subunits in growth plate chondrocytes. A: The expression of each subunit of nAChR. Total RNA was isolated from primary culture of human growth plate chondrocytes. The primers for each subunit are listed in Tables S1–S3. RT-PCR amplified products of alpha5, alpha7, beta1 and epsilon subunits of nAChR and GAPDH. B: Western blot analysis of alpha7 subunit of nAChR in primary chondrocyte cultures. NC: negative control (adipocyte), PC: positive control (PC-12 cell), GPC1,2: human growth plate chondrocyte derived from extra fingers of two individuals. C: Immunocytochemical analysis of alpha7 nAChR subunit in human growth plate chondrocytes. Primary chondrocytes were stained with alpha7 nAChR subunit-specific antibody. D: Immunohistochemical analysis of alpha7 nAChR subunit in tibia of E15.5 fetuses. Alpha7 nAChR are detected at resting, proliferating and pre-hypertrophic chondrocytes of murine growth plate.

To date, many epidemiological [1]–[5] and experimental [13] studies suggested that endochondral ossification is affected by cigarette smoking, especially by its major component, nicotine [15]. We thus assumed that nicotine may directly affect chondrocytes, a key player in endochondral ossification. To investigate whether the impact of nicotine on chondrocytes is specific, we studied the expression pattern of the specific receptor, nAChR. For screening of the existing subunits of nAChR, RT-PCR was performed with primers for each subunit of nAChR. Human growth plate chondrocytes expressed alpha5, alpha7, beta1 and epsilon subunits of nAChR (Figure 1A).

Among the detected subunits, only the alpha7 subunit can form a functional nAChR by forming a homopentameric receptor [17]. We thus tried to detect alpha7 subunit at a protein level. Western blot analysis revealed that chondrocytes produced alpha7 nAChR (Figure 1B). Immunocytochemical analysis also revealed that chondrocytes stained positive for alpha7 nAChR (Figure 1C). Moreover, the alpha7 subunit was detected at resting, proliferating and pre-hypertrophic chondrocytes of murine growth plate but not hypertrophic chondrocytes (Figure 1D). These results suggest that the growth plate chondrocytes in their non-hypertrophic stage express alpha7 homopentameric nAChR.

### Effect of nicotine on chondrocytes cultured in agarose gel

**Figure 2:**
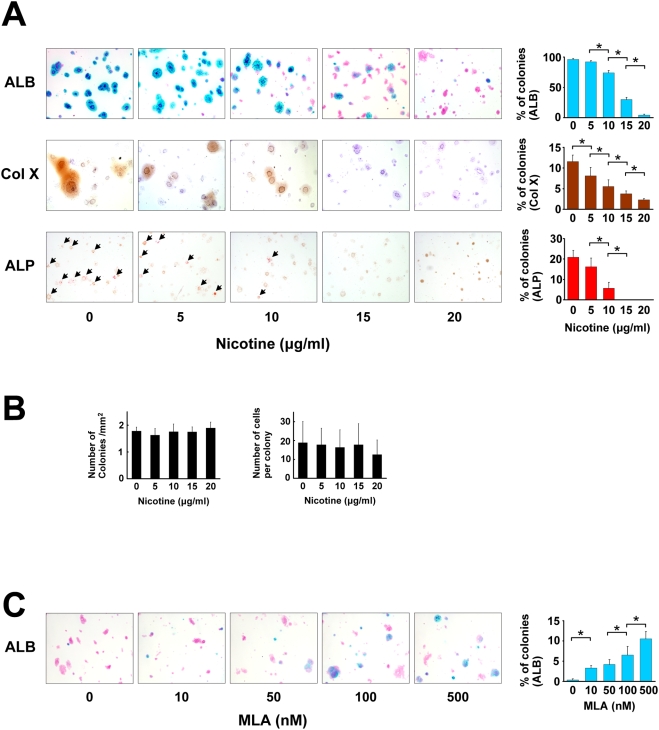
Effect of nicotine on growth plate chondrocytes in agarose gel. Growth plate chondrocytes were cultured in an agarose gel using the modified method previously described [28], and exposed to nicotine and MLA, a specific antagonist for alpha7 nAChR, at the indicated concentration. After three weeks of cultivation, suspension agarose was transferred to a glass slide and the following histological analyses were then performed. A: Microscopic appearance of chondrocyte colonies. From top to bottom: ALB (Alcian blue stain), Col X (immunocytochemistry by an anti-Col X antibody), ALP (enzyme cytochemistry of alkaline phosphatase). For ALB and Col X stain, the slides were counterstained with kernechtrot and hematoxylin, respectively. Percentage of ALB- stained, Col X- positive, and Alkaline phosphatase- positive colonies were counted (right panel, from top to bottom). All the ALP positive colonies in the panels are indicated by arrowheads. Nicotine concentration-dependently suppressed the percentage of the colonies stained with ALB, Col X, and ALP. *, statistically significant, P<0.02. B: Number of colonies and number of cells per colony. The number of colonies with a diameter greater than 50 µm (left panel) and cell number per colony (right panel) were counted on the ALB- stained agarose gel slides. C: Microscopic appearance of chondrocyte colonies stained with ALB. MLA reversed the decrease of ALB- positive matrix in a concentration-dependent manner under constant nicotine concentration (20 µg/ml). The percentage of ALB-positive colonies exceeded 10% by using 500 nM MLA. *, statistically significant, P<0.02.

To study the effect of nicotine on growth plate chondrocytes in vitro, two methods of suspension cultures, i.e., agarose gel culture and alginate bead culture, were employed. In agarose gel, the chondrocytes are initially embedded in the suspension layer solitarily. The chondrocytes then proliferate, differentiate, and aggregate to form a colony in the presence of ascorbic acid, and start to produce a matrix around themselves [18]. We applied the agarose gel culture to study the effect of nicotine on the proliferation and differentiation of growth plate chondrocytes in vitro. Nicotine was added to culture media for three weeks culture period. Nicotine decreased the percentage of colonies which produce matrix, as revealed by alcian blue (ALB) stains in a concentration-dependent manner (Figure 2A, upper panels). Similarly, nicotine suppressed Col X expression and enzyme activity of alkaline phosphatase (ALP) in a concentration-dependent manner (Figure 2A, middle and lower row panels). In contrast, nicotine did not affect colony density (Figure 2B, left panel) or the number of cells per colony (Figure 2B, right panel) which are indicators for cell proliferation. No nicotinic effect on cell proliferation was detected as assessed by immunohistochemistry using antibody to proliferating cell nuclear antigen (PCNA) (Figure S1). These results suggest that nicotine decreases the matrix synthesis and suppresses hypertrophic differentiation of growth plate chondrocytes, but has little effect on cell proliferation in vitro and vivo. To investigate if the nicotinic effect is mediated by alpha7 nAChR, we used MLA, the specific antagonist of alpha7 nAChR. MLA clearly reversed the effect, as assessed by ALB-stained colonies (Figure 2C), suggesting the involvement of alpha7 nAChR in the effect of nicotine on growth plate chondrocytes.

### Long-term (four months) effect of nicotine on growth plate chondrocytes in alginate beads

**Figure 3:**
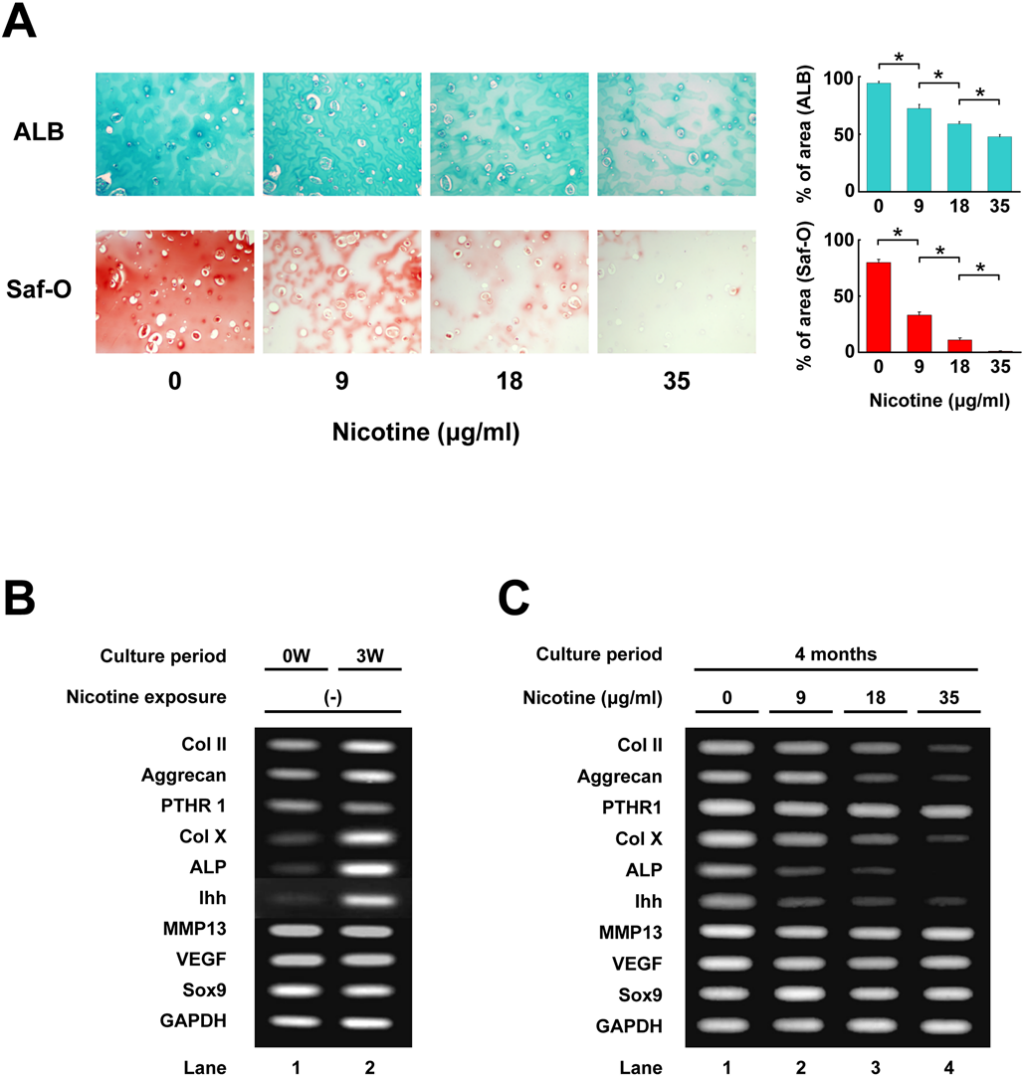
Long-term (four months) effect of nicotine on growth plate chondrocytes in alginate beads. Growth plate chondrocytes in alginate beads were exposed to the indicated concentration of nicotine for four months. A: Microscopic view of chondrocytes in alginate beads after four-months cultivation. Upper panels: ALB stain, lower panels: Safranin-O stain. Chondrocytes were surrounded by matrix which they secreted. Nicotine decreased the area stained with ALB or Safranin-O in a concentration-dependent manner. *, statistically significant, P<0.02. B: RT-PCR analysis of chondrocyte-specific gene expression in the chondrocytes at the start of cultivation (lane 1: 0W) and three weeks (lane 2: 3W). From top to bottom: genes for Col II, Aggrecan, parathyroid hormone receptor type 1 (PTHR1), Col X, alkaline phosphatase (ALP), Indian hedgehog (Ihh), matrix metalloproteinase type13 (MMP13), vascular endothelial growth factor (VEGF), Sox9 and GAPDH. C: RT-PCR analysis of chondrocyte-specific gene expression in chondrocytes embedded in alginate beads exposed to the indicated concentration of nicotine for four months. Expression of early stage matrix-gene (Col II and Aggrecan) and markers of hypertrophic chondrocytes (Col X, ALP and Ihh) increased after three weeks of cultivation (B). Nicotine decreased the expression of these genes in a concentration-dependent manner, but had little effect for the expression of MMP13, VEGF, and control genes (Sox9 and GAPDH) (C).

Different from the case with agarose gel, human chondrocytes hardly proliferate in alginate beads, maintaining chondrocyte properties for more than eight months [19]. Moreover, molecular analysis can be done easily compared with that in agarose gel, since chondrocytes can be recovered from beads by chelation of divalent ions with ethylenediamine tetraacetic acid (EDTA) followed by centrifugation. We investigated the long-term effect of nicotine on growth plate chondrocytes by employing alginate bead culture. Chondrocytes encapsulated in alginate beads remained viable during the culture period (four months) in their lacunae. Nicotine did not affect viability of the chondrocytes at any indicated concentration. Nicotine dose-dependently suppressed ALB- and Safranin-O-stained areas at four months (Figure 3A).

To investigate expression of chondrocyte-specific genes, we performed RT-PCR analysis on chondrocytes in alginate beads. Genes for collagen type II (Col II), Aggrecan, collagen type X (ColX), ALP, and indian hedgehog (Ihh) were up-regulated at three weeks after the start of alginate bead culture (Figure 3B). In contrast, genes for parathyroid hormone receptor type I (PTHR1), matrix metalloproteinase type 13 (MMP13), vascular endothelial growth factor (VEGF), and Sox9 were constitutionally expressed and their expression level remained unchanged. We then performed RT-PCR analysis to investigate the expression of chondrocyte-specific genes in chondrocytes treated by nicotine for four months. Nicotine dose-dependently decreased the expression of Col II, Aggrecan, Col X, ALP, and Ihh gene (Figure 3C). These findings suggest that nicotine suppresses matrix synthesis and hypertrophic maturation of chondrocytes in long-term culture using alginate beads.

### Functional calcium imaging

**Figure 4:**
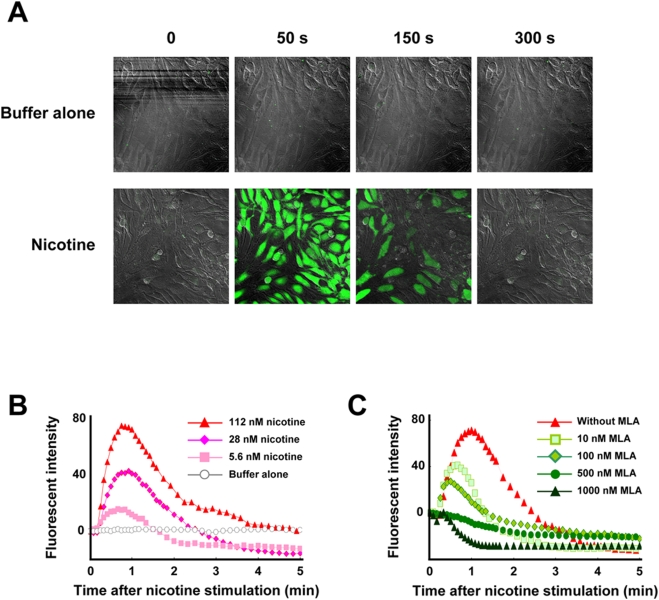
Calcium influx assay in primary chondrocyte culture. Nicotine-stimulated calcium signaling was investigated by the use of a fluorescent Ca^2+^ indicator. Primary chondrocyte cultures were stimulated by nicotine with or without MLA, the specific antagonist of alpha7 homomeric nAChR. A: Addition of assay buffer alone elicits no reaction (upper panels: negative control). Nicotine elicits a transient increase of intra-cellular calcium (lower panels). B: Nicotine elicits a transient increase of intra-cellular calcium in a concentration-dependent manner. C: MLA inhibits nicotine-induced calcium influx in a concentration-dependent manner. The cells were treated with MLA 30 min before nicotine stimulation.

To investigate the intracellular signals after nicotinic stimulation, we performed calcium imaging assay for primary chondrocyte cultures, since alpha7 nAChR has large Ca^2+^ permeabilities and also induces elevated intracellular free calcium by releasing intracellular calcium stores [17]. Nicotine elicited a transient increase of intra-cellular calcium (Figure 4A) in a concentration-dependent manner (Figure 4B). MLA, the specific antagonist of alpha7 nAChR, inhibited the calcium signals in a concentration-dependent manner (Figure 4C), implying that the effect of nicotine on chondrocytes is mediated through the alpha7 nAChR.

### Maternal nicotine exposure in wild-type mice

**Figure 5:**
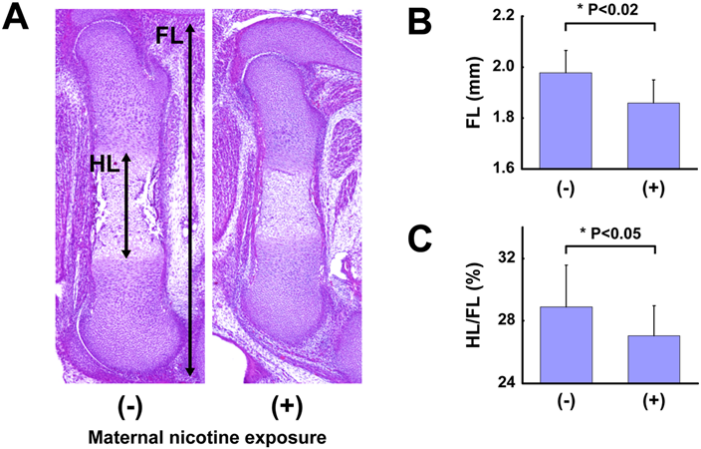
Maternal nicotine exposure in wild-type mice. Ovulation-induced pregnant mice were mated and were given drinking water with nicotine during pregnancy. At noon on gestational day 15, the fetuses were sacrificed, and their legs were histologically investigated. A: Skeletal growth estimated by measuring the femur length (FL) and the length of the hypertrophic zone of the femur (HL). B: FL (mm), C: HL/FL (%) of E15.5 fetuses whose mothers were given drinking water with or without nicotine. Nicotine significantly decreased FL and HL/FL.

To study the effect of nicotine on endochondral ossification in vivo, ovulation-controlled pregnant C57BL/6J mice were given drinking water with or without nicotine during pregnancy, and skeletal growth of their fetuses was observed. At noon on gestational day 15, fetuses were surgicallly obtained and their legs were sectioned for measurement of the femur length (FL) and the length of the hypertrophic zone of the femur (HL) (Figure 5A). There were no significant differences of the amount of water consumed between nicotine-exposed group and control group. Maternal nicotine exposure significantly reduced the FL (Figure 5B) and HL/FL (Figure 5C) of mice at embryonic day 15.5 (E15.5), suggesting that nicotine delayed endochondral ossification.

### Maternal nicotine exposure in alpha7 nAChR-disrupted mice

**Figure 6:**
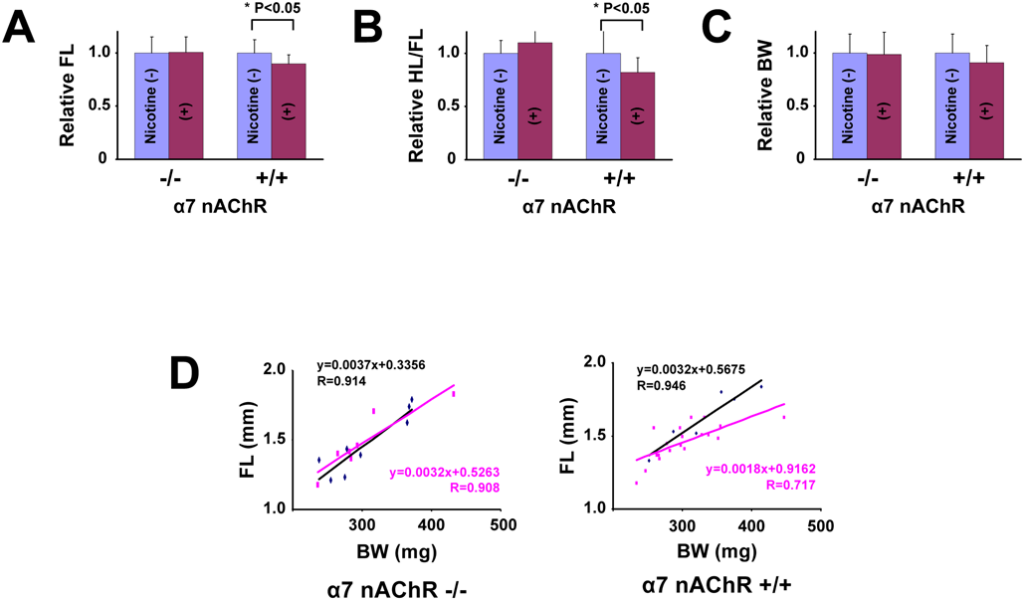
Maternal nicotine exposure in alpha7 nAChR-disrupted mice. A–C: FL, HL/FL, and body weight (BW) of alpha7 nAChR −/− and alpha7 nAChR +/+ E15.5 littermate fetuses. Alpha7 nAChR +/− female were mated with alpha7 nAChR +/− male, and given drinking water with or without nicotine during pregnancy. Relative FL, HL/FL, and BW were calculated, each value in mice that did not receive nicotine was regarded as equal to 1.0. Nicotine significantly reduced FL and HL/FL in alpha7 nAChR +/+ fetuses but not in alpha7 nAChR −/− fetuses (A,B). Nicotine did not significantly reduce BW in either genotype (C). D: Scatterplot and correlation between the FL and BW of mice with (red line) or without (black line) exposure to nicotine. In alpha7 nAChR +/+ fetus, Nicotine downwardly shifts the linear slope in alpha7 nAChR +/+ fetuses but not in alpha7 nAChR −/− fetuses.

To clarify an involvement of alpha7 nAChR in nicotine-induced delayed skeletal growth in vivo, we investigated the effect of maternal nicotine exposure on skeletal development of murine fetuses in which the alpha7 nAChR gene is disrupted. Maternal genotype is alpha7 nAChR +/− in this experiment (Figure 6), unlike the experiment using wild type mice (Figure 5, maternal genotype: alpha7 nAChR +/+), and littermate fetuses (alpha7 nAChR −/− and alpha7 nAChR +/+) were compared to exclude the effect of nicotine on maternal bodies. Nicotine significantly reduced FL and HL/FL in alpha7 nAChR +/+ fetuses but not in alpha7 nAChR −/− fetuses (Figure 6A, B). However, nicotine did not significantly affect body weight (BW) in both genotypes (Figure 6C). Besides, scatterplot and correlation between the FL and the BW revealed that nicotine downwardly shifted the linear slope in alpha7 nAChR +/+ fetuses but had no effect in alpha7 nAChR −/− fetuses (Figure 6D). These findings suggest that maternal nicotine exposure decreased the fetal endochondral ossification through the fetal alpha7 nAChR in vivo.

## Discussion

Alpha7 nAChR was originally identified as a subunit of neuronal nAChR, and has also been shown to be functional in both neuronal and non-neuronal, i.e., non-excitable cells such as lymphocytes, vascular endothelial cells, keratinocytes and bronchial epithelium [16]. In this study, we demonstrated the expression of the alpha7 subunit of nAChR at resting to pre-hypertrophic chondrocytes in murine growth plate and on a culture of human growth plate chondrocytes, and the involvement of alpha7 nAChR in nicotine-induced delayed skeletal growth. The novel findings of alpha7 nAChR in chondrocytes suggest that the effect of smoking on delayed skeletal growth is directly correlated with nicotinic action on chondrocytes.

### Direct effect of nicotine on human growth plate chondrocytes

Maternal nicotine exposure decreases the width of the hypertrophic zone of growth plate, increases apoptotic chondrocytes, and reduces the length of femur in rat [20]. Contrarily, nicotine has been shown to up-regulate glycosaminoglycan and collagen synthesis of human articular chondrocytes in vitro [21]. Cultured human growth plate chondrocytes derived from infant fingers serve as a good model for analyzing whether nicotine has direct action on growth plate chondrocytes. The present findings of nicotinic effect, i.e. decreasing matrix synthesis and suppressing hypertrophic differentiation but not proliferation on growth plate chondrocytes in vitro, indicate the direct effect of nicotine on growth plate chondrocytes. The findings are consistent with reports that maternal nicotine exposure has a negative effect on endochondral ossification in animals [13]. Besides, these findings are consistent, considering the fact that longitudinal skeletal growth is partly caused by matrix synthesis and hypertrophic differentiation of chondrocytes. Confirmation of the animal model using “human” chondrocytes is essential since certain chemicals, such as thalidomide, exhibit different effects in humans and rodents.

Differences of expression levels of the genes for Col X, ALP, Ihh, MMP13, and VEGF in alginate beads culture (Figure 3B, C) may attribute to differential regulation among hypertrophic markers. Expression of the Ihh, Col X, and ALP genes were down-regulated by nicotine and the MMP13 and VEGF genes remained unaffected. Alternatively, the difference could be a result of chondrocyte culture, that is, artifactual induction ex vivo, and the MMP13 and VEGF genes were indeed expressed at the start of alginate bead culture with chondrocytes at passage 1 (Fig. 3B, lane 1: “0 W”). In contrast, the Col X, ALP, and Ihh genes were appropriately regulated after three-dimensional culture (Figure 3B, lane 2: “3 W”; Figure 3C, lane 1: without exposure to nicotine), as is the case with gene regulation in the growth plate.

### Involvement of alpha7 nAChR in delayed endochondral ossification

The alpha7 nAChR-null mice exhibit normal development, including neural tissue, but alpha7 nAChR-null mice lack nicotinic currents in hippocampal neurons [22], and show abnormalities in late-stage keratinocyte development in the epidermis [23]. Lack of phenotypic abnormality in the femur of fetuses (Figure 5B) and adults indicates that ACh signaling through alpha7 nAChR has little involvement in the process of physiological skeletal growth. Results using MLA, the antagonist to alpha7 nAChR, strongly suggest the involvement of alpha7 nAChR in the nicotinic effect on chondrocytes. Such low-molecular weight substances may, however, have additional unclarified action in addition to any “specific” action. The proof of alpha7 nAChR involvement in delayed skeletal growth was strengthened by the in vivo experiments with alpha7 nAChR gene-disrupted mice. Especially so, considering the fact that maternal nicotine exposure caused delayed skeletal growth in only alpha7 nAChR +/+ fetuses compared with their alpha7 nAChR −/− littermates, fetal alpha7 nAChR but not maternal alpha7 nAChR is responsible for the mechanism of nicotine-induced delayed skeletal growth.

Since nicotine exposure has been reported to be epidemiologically and experimentally correlated with maternal effect, i.e., abnormal placental function and blood flow [10], the physiological and pathological function of alpha7 nAChR in growth plate was confirmed by comparing “littermates” of alpha7 nAChR (Figure 6B). This comparison confirms involvement of alpha7 nAChR on the fetus, and eliminates a possibility of maternal effect. Furthermore, decrease of relative femur length (Figure 6C, scatterplot and correlation, right panel, alpha7 nAChR +/+) and lack of nicotinic effect on body weight of alpha7 nAChR fetuses (Figure 6B, right panel, “BW”) by maternal nicotine exposure indicate a specific effect of nicotine on bone growth rather than a systemic effect. Therefore, the effect of smoking during pregnancy on skeletal growth may be attributed to this direct action of nicotine on growth plate chondrocytes, at least in part.

Our studies suggest that, from the large number of chemicals associated with cigarette smoking, nicotine may cause delayed skeletal growth and, indeed, amniotic fluid and breast milk both have higher concentrations of nicotine than maternal serum does [24]. In addition, metabolism of nicotine in the fetus and child is much slower than that in adults [25]. We therefore should pay close attention to the effect of smoking, regardless of being active or passive, on growth plate chondrocytes. This nicotinic effect may also extend to the delay of fracture repair or generation of non-union in adults, since the process of bone repair also partly depends on endochondral ossification.

## Materials and Methods

### Chondrocyte cultures

Human chondrocytes were isolated from epiphysis of extra fingers, which were surgically excised from patients with polydactyly. Ethical approval for tissue collection was granted by the Institutional Review Board of the National Research Institute for Child Health and Development, Tokyo, Japan (#88). Minced tissue was incubated for 1 h at 37°C in 0.08% trypsin in PBS, then for 6 h at 37°C in 0.2% collagenase type 1 (Wako, Osaka, Japan) in Dulbecco's Modified Eagle's medium (DMEM). The released cells were washed and resuspended in DMEM containing 10% fetal bovine serum (FBS, Sanko Junyaku Co., Tokyo, Japan, lot number: 27110307) and plated at a density of 1×10^6^ cells per 100 mm dish for primary monolayer cultures, or 1×10^6^ cells per 35 mm dish for calcium influx assay and immunocytochemical assay of nAChR. In each experiment, we used one lot of cultured chondrocytes from extra fingers obtained from four patients.

### RT-PCR for detection of nAChR subunit

Total RNA was prepared from epiphysis of extra fingers using Isogen (Nippon Gene) according to the manufacturer's recommendations. DNase-treated RNA was reverse transcribed in 20 µl of RT-PCR mix (50 mM Tris, pH 8.3, 3 mM MgCl_2_, 75 mM KCl, 50 mM dNTPs, 2.5 µM oligo(dT)_20_, 5 mM DTT, 2 U RNaseOUT and 10 U SuperScriptIII (Invitrogen) at 50°C for 1 h. The PCR was performed in a final volume of 50 µl containing 1 µl of the single strand cDNA product, 25 mM TAPS (pH 9.3), 50 mM KCl, 2.0 mM MgCl_2_, 1 mM β-mercaptoethanol , 200 µM dNTPs, and AmpliTaq Gold (Applied Biosystems) and 20 pmol of each forward (5′) and reverse (3′) primers (Table S1). For each experiment the housekeeping gene GAPDH was amplified with 25–35 cycles to normalize the cDNA content of the samples. The amplification was performed for 30 cycles, with other conditions following polymerase-producing manufacturer's recommendations. Human brain and skeletal muscle RNAs were purchased from Ambion (Austin, TX).

### Western blot analysis for detection of nAChR subunit

Total proteins were isolated from primary monolayer cultures using CelLyticTM-M Mammalian Cell Lysis/Extraction Reagent (Sigma). The proteins were separated by SDS-PAGE (Bio-Rad) in a 10% acrylamide gel, then blotted at 60 V for 2 h at 4°C onto a nitrocellulose membrane. Non-specific binding was blocked by incubation in TBS containing 10% BSA and 0.05% Tween-20. The membrane was subsequently incubated at 4°C overnight with the monoclonal antibody to nicotinic acetylcholine receptor, alpha7 subunit (Sigma, St-Louis, MO; product number: N 8158) diluted 1:3000. After rinsing, the membrane was incubated for 1 h at room temperature in horseradish peroxidase-conjugated rabbit anti-rat IgG antibody (Sigma; A 5795) at a dilution of 1:3000 in TBS containing 0.05% Tween-20. After rinsing, the membrane was immersed in ECL solution (GE Healthcare, Buckinghamshire, UK). Then, the blots were visualized by LAS-1000plus IDX2, the luminescent image analyzer (Fuji Photo Film, Japan).

### Immunocytochemical and immunohistochemical analysis

Immunocytochemical analysis was performed as previously described [26]. Briefly, dishes were incubated with antibody to alpha7 subunit of nAChR in PBS containing 1% BSA. As a methodological control, the primary antibody was omitted. After washing in PBS, dishes were incubated with horseradish peroxidase (HRP)-conjugated rabbit anti-rat IgG antibody. Staining was developed by using a solution containing diaminobenzidine and 0.01% H_2_O_2_ in 0.05 M Tris-HCl buffer, pH 6.7.

For immunohistochemical analysis, hind legs of E15.5 C57BL/6J mice were prepared, fixed in 4% paraformaldehyde phosphate buffer solution (Wako) overnight at 4°C, and embedded in paraffin. Immunohistochemical analysis was performed as previously described [27]. Briefly, slides were treated with 0.4% pepsin (DAKO) at 37°C for 30 min, incubated with primary antibody to alpha7 subunit of nAChR(Sigma, product number: N 8158) diluted 1:2000 in PBS containing 1% BSA at room temperature for 3 h, and incubated with simple mouse stain MAX-PO (RAT), a second antibody, at room temperature for 1 h. Staining was developed by using a solution containing diaminobenzidine and 0.01% H_2_O_2_ in 0.05 M Tris-HCl buffer, pH 6.7. Finally, slides were counterstained with hematoxylin.

### Agarose gel cultures

Chondrocytes were cultured in agarose-stabilized suspension using a modified method as previously described [28]. Primary monolayer cultures were trypsinized, re-suspended in agarose gel medium : DMEM/F-12 containing 10% FBS, 100 units/ml penicillin G, 100 mg/ml streptomycin, and 50 mg/ml ascorbate, to a concentration of 2×10^4^ cells/ml, then mixed with equal volume of 1% low-temperature melting agarose (Sigma-Aldrich, Steinheim, Germany) in agarose gel medium, giving a final concentration of 1×10^4^ cells/ml suspended in 0.5% low-temperature melting agarose in agarose gel medium (suspension agarose). Three milliliters of suspension agarose were added to 60 mm culture plates that were precoated with 2 ml of 1% autoclaved standard agarose (Bio-Rad, Hercules, CA). The gel was allowed to solidify at 4°C before addition of agarose gel medium. Then, culture plates were placed in a 37°C, 5% CO_2_ humidified incubator for 21 days, and medium containing indicated concentration of nicotine was replaced once at the beginning of the week. After 21 days, suspension agarose was transferred to a glass slide, and placed on a plate warmer at 50°C with a covering of positively-charged nylon membranes (Roche, Mannheim, Germany). The slides were completely dried in a incubator at 42°C overnight, and fixed in 4% paraformaldehyde for 15 min, and stained with ALB to identify colonies producing glycosaminoglycans and to observe histologically. Colonies were defined as a cluster of cells with a diameter greater than 50 µm. ALP activity was determined in non-fixed agarose slide by Histofine, ALP substrate kit (Nichirei, Tokyo, Japan) following the manufacturer's product information. Type 10 collagen expression was also determined in the agarose slide using specific monoclonal antibody (Sigma; product number: C7974). The slide was fixed in acetone (Nacalai Tesque, Kyoto, Japan) at room temperature for 20 min. Non-specific binding was blocked with 2.5% normal rabbit serum (DakoCytomation, Glostrup, Denmark) in PBS containing 1% BSA and 1% Triton X-100. Slides were incubated for 6 h at room temperature with primary antibody, diluted 1:2000 in PBS containing 1% BSA. Bound antibody was detected by HRP-conjugated polyclonal rabbit anti-mouse IgM antibody (Dako, Glostrup, Denmark; product number: P 0260) diluted 1:100 in PBS at room temperature for 30 min. Peroxidase activity was visualized with diaminobenzidine tetrahidrochloride plus 0.03% H_2_O_2_, and slide was counterstained with hematoxylin.

### Alginate bead cultures

Chondrocytes were cultured in alginate beads following the method described by De Ceuninck et al. Primary monolayer cultures were trypsinized, washed, and centrifuged. The isolated chondrocytes were suspended at a concentration of 2×10^6^ cells/ml in a 1.25% alginate in 0.15 M NaCl. The cell suspension was slowly expressed through a 21 gauge needle and dropped into a 102 mM CaCl_2_ solution. The beads with approximately 25,000 cells/bead were allowed to polymerize for 10 min and washed three times with 0.15 M NaCl, followed by two washes in DMEM/F12. The beads were then transferred to medium (200 beads/10 ml/60 mm culture dish): DMEM/F-12 containing 10% FBS, 50 µg/ml ascorbate, 100 units/ml penicillin G, 100 mg/ml streptomycin. The beads were cultured at 37°C in a 5% CO_2_ humidified incubator for four months, and medium with or without nicotine was replaced twice weekly. The beads were transferred to new dishes every other week to avoid the formation of monolayer cultures on the bottom of the dish by chondrocytes escaping from the beads.

For histological analysis, the beads were fixed in 4% paraformaldehyde, 0.1 M cacodylate buffer, pH 7.4, containing 10 mM CaCl_2_ for 4 h at room temperature, and then washed overnight at 4°C in 0.1 M cacodylate buffer, pH 7.4, containing 50 mM BaCl_2_. The beads were dehydrated through alcohols and embedded in paraffin. The sections were routinely stained with ALB and safranin-O.

For RT-PCR analysis, chondrocytes were separated from the beads by incubating the beads in dissolution solution (at a ratio of 200 µl/bead), containing 55 mM EDTA, for 5 min and centrifuged. Total RNA was isolated by using RNeasy (Qiagen) following manufacturer's instructions, and was converted to cDNA by same method as described above. The sequences of PCR primers of human chondrocyte-related gene are listed in Table S2. PCR was performed in a final volume of 50 µl containing 2 µl of the single strand cDNA product (10 ng/µl), 10 mM Tris-HCl (pH8.3), 50 mM KCl, 1.5 mM MgCl_2_, 200 µM dNTPs, 1.25 U Taq (Takara), and 20 pmol of each forward (5′) and reverse (3′) primers.

### Calcium imaging

Primary monolayer cultures in 35 mm glass-bottomed plates were prepared. At near confluence, measurement was done by using Fluo-4 NW calcium assay kit (Molecular Probes, product number: F36206) following the manufacturer's product information. In short, the cells were incubated in dye loading solution containing 2.5 mM probenecid at 37°C for 30 min, then at room temperature for an additional 30 min before nicotine stimulation. The fluorescence was measured in LSM 510 (Carl Zeiss) with the settings appropriate for argon laser. Nicotine and its antagonists were prepared as a solution in assay buffer. If antagonists were used, they were added 30 min prior to nicotine stimulation.

### Maternal nicotine exposure in wild-type mice

Three-month-old pregnant mice were purchased at day 1 of pregnancy from Sankyo Laboratories (Tokyo, Japan). The mice were given drinking water containing 2% sucrose (Wako, Osaka, Japan) with or without nicotine (hydrogen tartrate salt; Sigma-Aldrich, St. Louis, MO). Nicotine was added to the sucrose solution starting at an initial concentration of 25 µg/ml to the treatment group mice. This was increased to 50 µg/ml on days 3 to 4, 100 µg/ml after day 5. The control mice were given only sucrose solution as a drinking water. The pregnant mice were sacrificed at noon on gestational day 15. The embryo were immediately weighed, and the legs were immediately removed and fixed in 4% paraformaldehyde phosphate buffer solution (Wako) for 24 h. Then, the legs were dehydrated through alcohols, embedded in paraffin, and sections were stained with Hematoxylin and Eosin for histological analysis.

### Maternal nicotine exposure in alpha7 nAChR-disrupted mice

B6.129S7-Chrna7<tm1Bay>/J, the alpha7 nAChR +/− mice were obtained from Charles River Laboratories Japan. Ten- to 12-week old alpha7 nAChR +/− mice were mated, and pregnant mice were given sucrose solution with or without nicotine. The fetuses were obtained and analyzed as in the case of wild-type C57BL/6J mice, as described above. The alpha7 nAChR genotype was determined by means of PCR reaction with the specific primers (Table S3).

### Statistics

The results of the quantative assays were expressed as mean±S.D. Significance was determined with Student's *t* test and ANOVA. All experiments were replicated twice.

## Supporting Information

Figure S1 In vivo chondrocytic proliferation assay. A: Paraffin section of the femur of E15.5 C57BL/6J mice immunohistochemically stained with antibody to PCNA. Proliferative chondrocytes extensively stained positive for PCNA regardless of maternal nicotine exposure. B: Percentage of PCNA-positive cells in chondrocytes of the proliferative zone. There is no significant difference between the two groups. Hind legs of E15.5 C57BL/6J mice were prepared, fixed in 4% paraformaldehyde phosphate buffer solution (Wako) overnight at 4°C, and embedded in paraffin. After deparaffinization, slides were autoclaved in 0.01 M citrate buffer (pH 6.0) for 10 min, incubated with primary antibody to PCNA (DAKO: PC10) diluted 1:400 in PBS containing 1% BSA at room temperature for 3 h, and incubated with polyclonal rabbit anti-mouse immunoglobrins/HRP (DAKO: P260) at room temperature for 1 h. Staining was undertaken using a solution containing diaminobenzidine and 0.01% H_2_O_2_ in 0.05 M Tris-HCl buffer at pH 6.7, followed by counterstaining with hematoxylin.
(0.06 MB PDF)

Table S1 Primers for nAChR subunit genes
(0.03 MB PDF)

Table S2 Primers for chondrocyte specific genes
(0.01 MB PDF)

Table S3 Primers for genotypying alpha7 nAChR gene
(0.01 MB PDF)
