# Supplementary figures and images for: Nicotine Acts on Growth Plate Chondrocytes to Delay Skeletal Growth through the α7 Neuronal Nicotinic Acetylcholine Receptor
Source: PLoS One. 2008 Dec 16;3(12):e3945. doi: 10.1371/journal.pone.0003945 (PMC2596484; doi:10.1371/journal.pone.0003945)

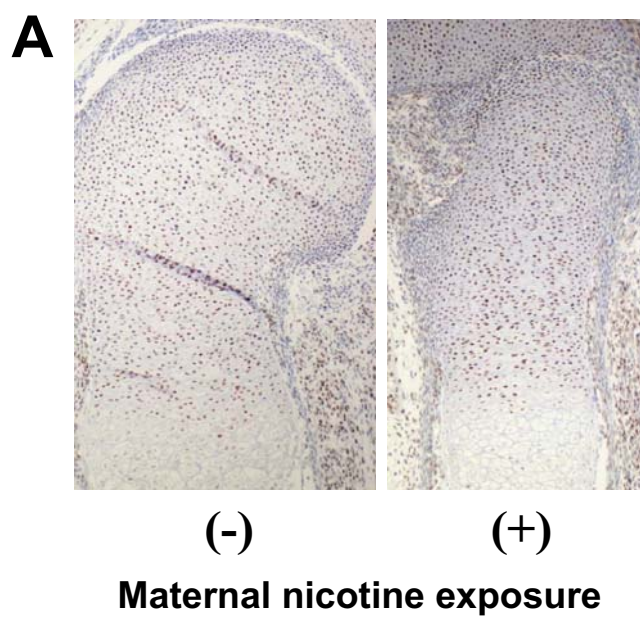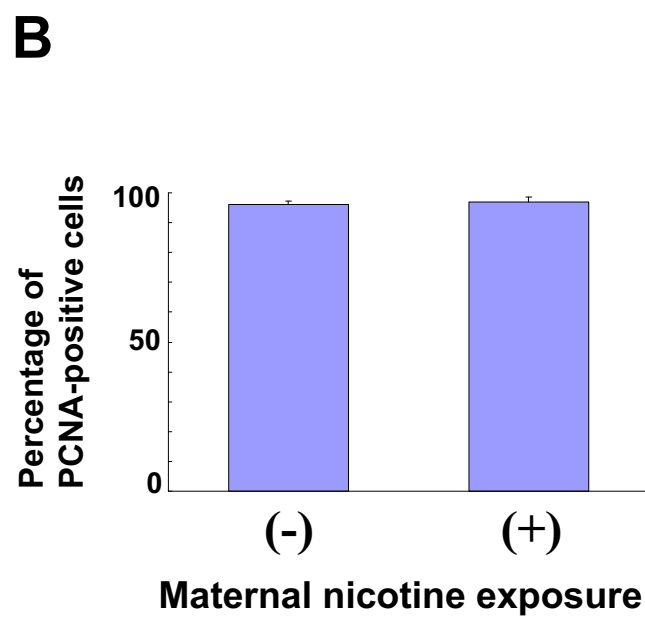

**Figure S1**

Supplement: Figure S1 — In vivo chondrocytic proliferation assay. A: Paraffin section of the femur of E15.5 C57BL/6J mice immunohistochemically stained with antibody to PCNA. Proliferative chondrocytes extensively stained positive for PCNA regardless of maternal nicotine exposure. B: Percentage of PCNA-positive cells in chondrocytes of the proliferative zone. There is no significant difference between the two groups. Hind legs of E15.5 C57BL/6J mice were prepared, fixed in 4% paraformaldehyde phosphate buffer solution (Wako) overnight at 4°C, and embedded in paraffin. After deparaffinization, slides were autoclaved in 0.01 M citrate buffer (pH 6.0) for 10 min, incubated with primary antibody to PCNA (DAKO: PC10) diluted 1:400 in PBS containing 1% BSA at room temperature for 3 h, and incubated with polyclonal rabbit anti-mouse immunoglobrins/HRP (DAKO: P260) at room temperature for 1 h. Staining was undertaken using a solution containing diaminobenzidine and 0.01% H2O2 in 0.05 M Tris-HCl buffer at pH 6.7, followed by counterstaining with hematoxylin. (0.06 MB PDF) [file pone.0003945.s001.pdf]
