# Supplementary material for: Nicotine Acts on Growth Plate Chondrocytes to Delay Skeletal Growth through the α7 Neuronal Nicotinic Acetylcholine Receptor
Source: PLoS One. 2008 Dec 16;3(12):e3945. doi: 10.1371/journal.pone.0003945 (PMC2596484; doi:10.1371/journal.pone.0003945)
Supplement: Table S1 — Primers for nAChR subunit genes (0.03 MB PDF) [file pone.0003945.s002.pdf]

Table S1. primers for nAChR subunit genes

Gene product	Primer
nAchR-α1-for	CAGAAAATGAGCTGAGCTTAGC
nAchR-α1-rev	CTAAAGAGGGTTCACTCTTCAGG
nAchR-α2-for	GTGGAGGAGGAGGACAGA
nAchR-α2-rev	CTTCTGCATGTGGGGTGATA
nAchR-α3-for	CAGAGTCCAAAGGCTGCAAG
nAchR-α3-rev	AGAGAGGGACAGCACAGCAT
nAchR-α4-for	CTCACCGTCCTTCTGTGTC
nAchR-α4-rev	CTGGCTTTCTCAGCTTCCAG
nAchR-α5-for	CTTCACACGCTTCCCAAACT
nAchR-α5-rev	CTTCAACAACCTCACGGACA
nAchR-α6-for	TCCATCGTGGTGACTGTGT
nAchR-α6-rev	AGGCCACCTCATCAGCAG
nAchR-α7-for	GTACGCTGGTTTCCCTTTGA
nAchR-α7-rev	CCACTAGGTCCCATTCTC
nAchR-α9-for	GAAAGCAGCCAGGAACAAAG
nAchR-α9-rev	GCACTTGGCGATGTACTCAA
nAchR-α10-for	ACACAAGTGCCCTGAGACCT
nAchR-α10-rev	TCCCATCGTAGGTAGGCATC
nAchR-β1-for	GTCTCAAACTGTGGGTTTCA
nAchR-β1-rev	GCACAGGTGCTGACCTATAG
nAchR-β2-for	ACTGGAAGTACGTCGCC
nAchR-β2-rev	CACTACTGTGCAGCAGAGG
nAchR-β3-for	ATTACACCTTAGACCTGACATCTGG
nAchR-β3-rev	GTAGAACAAGCACATGCATTTG
nAchR-β4-for	CTGAATGCCTTGGAGGG
nAchR-β4-rev	GCATAGTAGGTGCTGCTACG
nAchR-δ-for	GGAGCCAGGAGACAGCAG
nAchR-δ-rev	CCTGTCTTGATTTCAGGGGA
nAchR-ε-for	CATTATGATCCCTTCCCCCT
nAchR-ε-rev	GCTTTCTGGAAGACTGGCAC
nAchR-γ-for	CTCACCAGACTGAGCCAACC
nAchR-γ-rev	ACTGCAGCAGGACACTAGCC
GAPDH-for	CGTCTTCACCACCATGGAGA
GAPDH-rev	CGGCCATCACGCCACAGCTT
