# Supplementary material for: Nicotine Acts on Growth Plate Chondrocytes to Delay Skeletal Growth through the α7 Neuronal Nicotinic Acetylcholine Receptor
Source: PLoS One. 2008 Dec 16;3(12):e3945. doi: 10.1371/journal.pone.0003945 (PMC2596484; doi:10.1371/journal.pone.0003945)
Supplement: Table S2 — Primers for chondrocyte specific genes (0.01 MB PDF) [file pone.0003945.s003.pdf]

Table S2. primers for chondrocyte specific genes

Gene product	Primer
AGC79-for	TACACTGGCGAGCACTGTAAC
AGC79-rev	CAGTGGCCCTGGTACTTGTT
ALP-for	AACACCACCCAGGGGAAC
ALP-rev	GGTCACAATGCCCACAGATT
COL2A1-for	TGTCAGGGCCAGGATGTC
COL2A1-rev	ATCATTATACCTCTGCCCATCC
COL10A1-for	CACCTTCTGCACTGCTCATC
COL10A1-rev	GGCAGCATATTCTCAGATGGA
IHH-for	TGCATTGCTCCGTCAAGTC
IHH-rev	CCACTCTCCAGGCGTACCT
MMP13-for	CCAGTCTCCGAGGAGAAACA
MMP13-rev	AAAAACAGCTCCGCATCAAC
PTHR1-for	CCTGAGTCTGAGGAGGACAAG
PTHR1-rev	CACAGGATGTGGTCCCATT
SOX9-for	GTACCCGCACTTGCACAAC
SOX9-rev	TCGCTCTCGTTCAGAAGTCTC
VEGF22-for	CATTGGAGCCTTGCCTTG
VEGF22-rev	ATGATTCTGCCCTCCTCCTT
GAPDH-for	AGCCACATCGCTCAGACA
GAPDH-rev	GCCCAATACGACCAAATCC
COL1A1-for	GGGATTCCCTGGACCTAAAG
COL1A1-rev	GGAACACCTCGCTCTCCAG
