# Supplementary material for: Nicotine Acts on Growth Plate Chondrocytes to Delay Skeletal Growth through the α7 Neuronal Nicotinic Acetylcholine Receptor
Source: PLoS One. 2008 Dec 16;3(12):e3945. doi: 10.1371/journal.pone.0003945 (PMC2596484; doi:10.1371/journal.pone.0003945)
Supplement: Table S3 — Primers for genotypying alpha7 nAChR gene (0.01 MB PDF) [file pone.0003945.s004.pdf]

Table S3. primers for genotypying alpha7 nAChR gene

Gene product	Primer
alpha7 KO 1002	CCTGGTCCTGCTGTGTTAAACTGCTTC
alpha7 KO 1003	CTGCTGGGAAATCCTAGGCACACTTGAG
alpha7 KO 1004	GACAAGACCGGCTTCCATCC
